# Prognostic value of apparent diffusion coefficient in neuroendocrine carcinomas of the uterine cervix

**DOI:** 10.7717/peerj.15084

**Published:** 2023-03-31

**Authors:** Jian Chen, Ning Ma, Mingyao Sun, Li Chen, Qimin Yao, XingFa Chen, Cuibo Lin, Yongwei Lu, Yingtao Lin, Liang Lin, Xuexiong Fan, Yiyu Chen, Jingjing Wu, Haixin He

**Affiliations:** 1 Department of Gynecology, Clinical Oncology School of Fujian Medical University, Fujian Cancer Hospital, Fuzhou, Fujian, China; 2 Department of Radiology, Clinical Oncology School of Fujian Medical University, Fujian Cancer Hospital, Fuzhou, Fujian, China; 3 Department of Clinical Nutrition, Fujian Provincial Hospital, Fuzhou, Fujian, China; 4 College of Finance, Fujian Jiangxia University, Fuzhou, Fujian, China; 5 Department of Drug Clinical Trial Institution, Clinical Oncology School of Fujian Medical University, Fujian Cancer Hospital, Fuzhou, Fujian, China; 6 Department of Medical Record, Clinical Oncology School of Fujian Medical University, Fujian Cancer Hospital, Fuzhou, Fujian, China; 7 Department of Pathology, Clinical Oncology School of Fujian Medical University, Fujian Cancer Hospital, Fuzhou, Fujian, China

**Keywords:** Neuroendocrine carcinoma of the uterine cervix, Apparent diffusion coefficients, Prognostic factors, Diffusion-weighted imaging

## Abstract

**Objectives:**

This research was designed to examine the associations between the apparent diffusion coefficient (ADC) values and clinicopathological parameters, and to explore the prognostic value of ADC values in predicting the International Federation of Gynecology and Obstetrics (FIGO) stage and outcome of patients suffering from neuroendocrine carcinomas of the uterine cervix (NECCs).

**Methods:**

This retrospective study included 83 patients with NECCs, who had undergone pre-treatment magnetic resonance imaging (MRI) between November 2002 and June 2019. The median follow-up period was 50.7 months. Regions of interest (ROIs) were drawn manually by two radiologists. ADC values in the lesions were calculated using the Functool software. These values were compared between different clinicopathological parameters groups. The Kaplan–Meier approach was adopted to forecast survival rates. Prognostic factors were decided by the Cox regression method.

**Results:**

In the cohort of 83 patients, nine, 42, 23, and nine patients were in stage I, II, III, and IV, respectively. ADC_mean_, ADC_max_, and ADC_min_ were greatly lower in stage IIB–IVB than in stage I–IIA tumours, as well as in tumours measuring ≥ 4 cm than in those < 4 cm. ADC_mean_, FIGO stage, and age at dianosis were independent prognostic variables for the 5-year overall survival (OS). ADC_min_, FIGO stage, age at diagnosis and para-aortic lymph node metastasis were independent prognostic variables for the 5-year progression-free survival (PFS) in multivariate analysis. For surgically treated patients (*n* = 45), ADC_max_ was an independent prognostic parameter for both 5-year OS and 5-year PFS.

**Conclusions:**

ADC_mean_, ADC_min_, and ADC_max_ are independent prognostic factors for NECCs. ADC analysis could be useful in predicting the survival outcomes in patients with NECCs.

## Introduction

Neuroendocrine carcinomas of the cervix (NECCs), an infrequent but highly invasive form of cervical cancer, represent less than 5% of cervical carcinomas (Gardner, Reidy-Lagunes & Gehrig, 2011; Li et al., 2020; Lin et al., 2020; Satoh et al., 2014). According to the World Health Organization classification, NECCs are divided into small cell neuroendocrine carcinomas (SCNECs) and large cell neuroendocrine carcinomas (LCNECs) (Satoh et al., 2014). Owe to the high incidence of early lymph node involvement and distant metastasis, the outcome of patients with NECCs is worse than those with other subtypes of cervical cancer. Radical surgery and chemoradiation are recommended as the primary treatment for patients with early- and advanced-stage disease, respectively (Bhatla et al., 2019). Due to the rarity of NECCs, most studies on NECCs are reported in small samples or are case reports (McCann et al., 2013; Yuan et al., 2015). Therefore, the prognostic parameters and treatment of NECCs are controversial. Advanced FIGO stage, lymph node involvement, large tumor size, older age and lymphovascular invasion have been reported to be associated with poor prognosis (Chen et al., 2021; Gadducci, Carinelli & Aletti, 2017). However, the described factors play a limited role in predicting the prognosis of NECCs.

MRI is a great tool for diagnosing and staging cervical tumours due to its high soft tissue resolution. On T1- and T2-weighted images (T1WI and T2WI, respectively) as well as contrast-enhanced images, MRI can show both morphologic and signal intensity properties (Nakamura et al., 2012). Diffusion-weighted imaging (DWI), a functional imaging technology, quantifies the free movement of water molecules (Brownian molecular movement) through ADC values (Bruix & Llovet, 2002; Fan et al., 2020). ADC describes the velocity and scope of molecular diffusion movements in various directions (Liang et al., 2016). Moreover, ADC values provide useful information about tumour aggressiveness, subtype characterisation, and treatment responses taking into consideration the limiting barriers in tissue compartments (De Robertis et al., 2018; Gu et al., 2019; Meyer et al., 2019; Perucho et al., 2020; Zou et al., 2019). Schob et al. (2017a) found that ADC value were useful in predicting lymphatic metastasis, and proliferative activity in thyroid cancer. In gastric cancer, Liu et al. (2014) demonstrated that ADC analysis is helpful to assess the pre-treatment T and N staging. To date, few studies have discussed the application of MRI in NECCs. Duan et al. (2016) found that NECCs are characterized by lower ADC values and homogeneous lesion texture on MRI images. However, no studies have reported the utility of ADC values in predicting the outcomes of patients with NECCs. In this study, we examined the associations between maximum, mean, and minimum ADC values (ADC_max_, ADC_mean_ and ADC_min_, respectively) and clinicopathological parameters, as well as the prognostic value of ADC values in predicting the stage and outcome in patients with NECCs, in a retrospective review of 83 patients. We also assessed the accuracy of MRI in the diagnosis of NECCs.

## Materials & Methods

### Patients and treatment

The research was approved by the Ethics Committee of Fujian Medical University Cancer Hospital (Reference No: K2021-043-01). Between November 2002 and June 2019, the clinicopathological information of 172 patients with pathological confirmed NECCs who received treatment at Fujian Medical University Cancer Hospital, were reviewed. All histologic slides were reviewed by two experienced pathologists to confirm the diagnosis of NECCs. The requirement for informed consent was waived due to the retrospective nature of this study. The following were the standards for inclusion: (1) those with pathological confirmed NECCs, (2) those who underwent pre-treatment abdominal and pelvic MRI in our centre, and (3) those who received treatment in our centre and had complete medical records. The exclusion standards were as shown: (1) presence of other concurrent malignancies, (2) history of cancer, (3) those who refused or discontinued treatment, and (4) lost to follow-up. Finally, 83 patients were recruited in the group.

### MRI imaging

A 1.5T MRI system (GE Signa HDxT) was used. Before the examination, the patients were required to drink enough water to fill their bladder to a moderate level. The MRI scan extended from the renal hilum to the perineum. Routine abdominal and pelvic MRI including the following sequences were acquired as follows: (1) sagittal T2WI: fast spin-echo (FSE) sequence, repetition time (TR)/echo time (TE), 4760/104 ms; matrix size, 320 × 192; field of view (FOV), 24 cm; slice thickness/intersection gap, 5/1 mm; (2) axial T2WI: TR/TE, 4320/105 ms; matrix size, 320 × 192; FOV, 24 cm; slice thickness/intersection gap, 5/1 mm; (3) axial T1WI: fast FSE sequence; TR/TE, 600/7.7 ms; matrix size, 320 × 256; FOV, 48 cm; slice thickness/intersection gap, 7/1 mm. The protocols for axial DWI (*b* = 0, 800 s/mm^2^) were as follows: TR, 4225 ms, TE, minimum time; matrix size, 128 × 128; FOV, 38 cm; slice thickness/intersection gap, 7/1 mm.

### Imaging analysis

**Figure 1:**
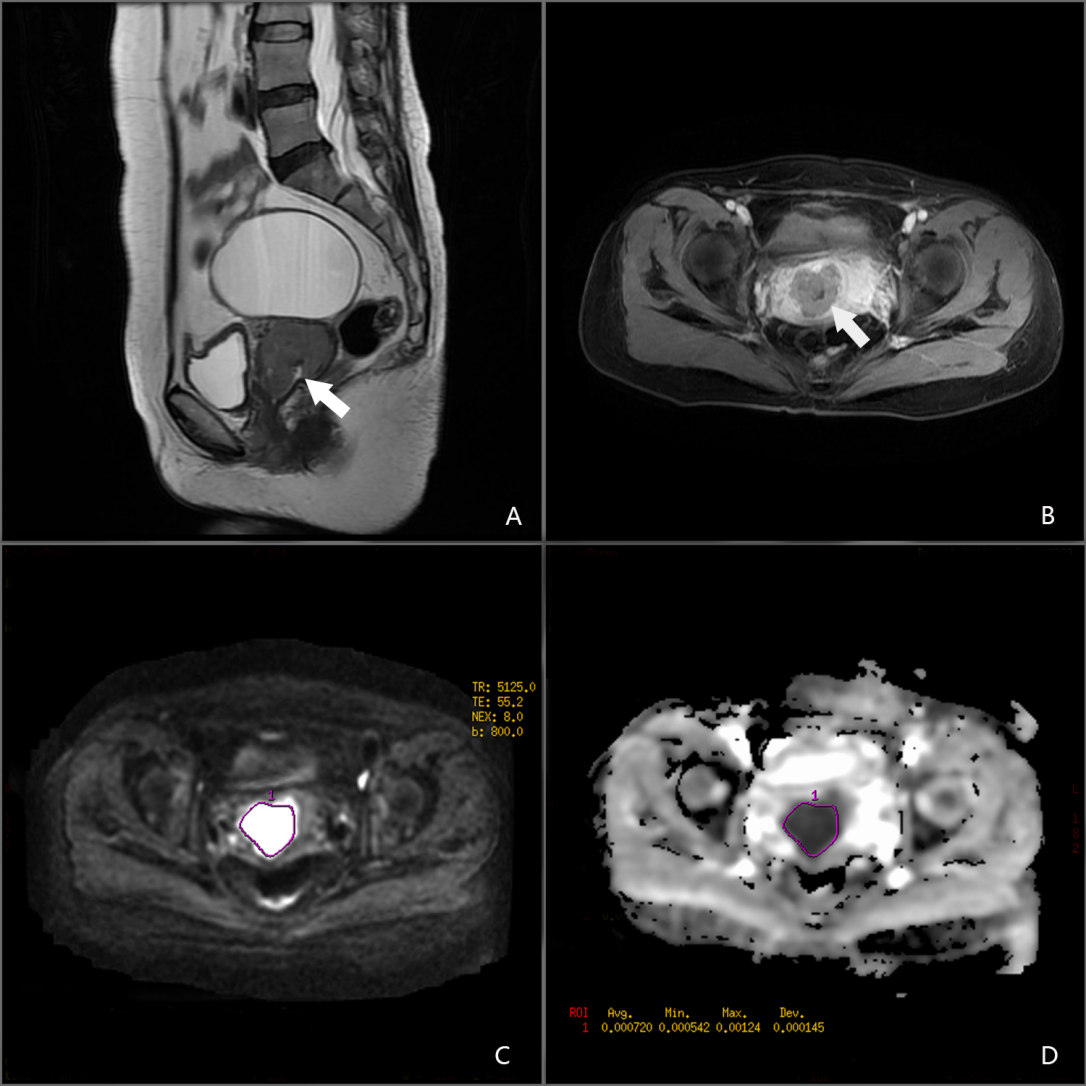
A 45-year-old female patient with NECC. (A) Sagittal T2-weighted image. (B) Axial T1-enhanced image. (C) DWI in *b* = 800. (D) ADC map.

All images were retrieved from the local picture archiving and communication systems. Two radiologists analysed the MR images in consensus (N.M. and X.F.C, with 8 and 12 years of experience in gynaecologic imaging, respectively). The two radiologists agreed on the criteria for determining the tumour size, vaginal extension, parametrial extension, and lymph node metastasis according to the FIGO 2018 staging and the guidance of the European Society of Urogenital Radiology (Balleyguier et al., 2011; Bhatla et al., 2018). They were blinded to the patients’ information. The ADC map was constructed automatically using the Functool software on the Advantage Workstation(AW 4.2 version, GE, US. https://www.medicalexpo.com/product-manufacturer/ge-mri-system-15892-438.html). Regions of interest (ROIs) were manually drawn along the margin of the lesions showing maximal tumour size on axial DWI images. The ROI did not include parts of the tumour that were cystic, necrotic, or haemorrhagic. ADC values in the lesions were calculated using the software (Fig. 1).

### Treatment

Since this was a retrospective study, therefore, the treatment plans of most cases were largely dependent on FIGO 2008 guidelines. Surgery was the primary treatment for patients in the early stage (FIGO stage I–IIA), and chemoradiation was applied for patients in the advanced stage (FIGO stage III–IV). For patients with IIB stage, whether to undergo surgery after neoadjuvant therapy or received chemoradiation treatment depends on the doctor’s judgment. According to the postoperative pathology report, adjuvant therapy was performed if there were risk factors such as lymph node metastases, positive margin, deep stromal invasion, lymphovascular invasion, parametrial invasion, and perineural invasion. Finally, 45 patients received surgery and 38 patients received chemoradiation or only chemotherapy. Of the 80 patients who received chemotherapy, 38 received etoposide and cisplatin/carboplatin (EP regimen), and 34 received paclitaxel and cisplatin/carboplatin (TP regimen). The other eight patients received bleomycin, ifosfamide, and cisplatin (one case); paclitaxel, etoposide, and cisplatin (three cases); docetaxel and platinum (two cases); and TP and EP successively (two cases).

### Statistical analysis

ADC values are shown as mean ± SD. The normality of all the data was tested by applying the Kolmogorov–Smirnov test. The Student’s *t*-test or the Mann–Whitney U test was adopted to compare the ADC values among different tumour groups. Applying the maximum Youden’s index, the ROC curve was used to estimate the parameter cutoff values. The Kaplan–Meier and Cox regression methods were adopted to calculate prognostic factors for OS and PFS. The multivariate analysis further investigated prognostic parameters (*p* values <0.1) from the univariate analysis. *P* value <0.05 was regarded statistically significant. The SPSS version 24.0 statistical software (SPSS Inc. Chicago, IL, USA, http://www.spss.com) was employed to perform all statistical analyses.

## Results

### Clinicopathologic characteristics of the patients

The clinicopathological characteristics of the patients are listed in Table 1. Eighty-three patients were enrolled; their ages ranged from 25 to 78 years, and the average age was 49.2 years. The average size of the cervical tumour was 4.7 cm. On the basis of the FIGO 2018 staging system, nine, 42, 23, and nine patients were in stage I, II, III, and IV, respectively. There were 80 and three cases of SCNEC and LCNEC, respectively. Pure histology was documented in 68.7% (57/83) of the patients. The mixed histology patterns included adenocarcinoma (16/83 patients; 19.3%), squamous cell carcinoma (7/83 patients; 8.4%), and adenosquamous carcinoma (3/83 patients; 3.6%). In the cohort of 83 patients, the mean values of ADC_max_, ADC_mean_, and ADC_min_ were 0.969, 0.750 and 0.632 (×10^−3^ mm^2^/s), respectively.

**Table 1:** Patient characteristics (*N* = 83).

Variables	Number(%)
Hystological type	
Small cell neuroendocrine carcinoma	80(96.4%)
Large cell neuroendocrine carcinoma	3(3.6%)
Histological homology	
Pure	57(68.7%)
Mix with squamous	7(8.4%)
Mix with adenocarcinoma	16(19.3%)
Mix with adenosquamous carcinoma	3(3.6%)
FIGO stage(2018)	
I	
IB2	6(7.2%)
IB3	3(3.6%)
II	
IIA1	13(15.7%)
IIA2	13(15.7%)
IIB	16(19.3%)
III	
IIIA	3(3.6%)
IIIC1	15(18.1%)
IIIC2	5(6.0%)
IV	
IVB	9(10.8%)
Lymph node metastasis	
Pelvic only	19(22.9%)
Pelvic and para-aortic	8(9.6%)
Negative	56(67.5%)
Primary treatment	
Surgery + adjuvant therapy	12(14.5%)
NACT + surgery ± adjuvant therapy	31(37.3%)
Surgery alone	2(2.4%)
CCRT + CT	30(36.1%)
CT alone	8(9.6%)
Chemotherapy regimen	
EP	38(45.8%)
TP	34(41.0%)
Other regimens	8(9.6%)
Without chemotherapy	3(3.6%)
Age, years(mean ± SD)	49.2 ± 10.5
Tumor size,cm(mean ± SD)	4.7 ± 1.9
ADC_*mean*_(10^−3^mm^2^/s,mean ± SD)	0.750 ± 0.293
ADC_max_ (10^−3^mm^2^/s,mean ± SD)	0.969 ± 0.284
ADC_min_(10^−3^mm^2^/s,mean ± SD)	0.632 ± 0.293

**Notes.**

Abbreviations FIGO International Federation of Gynecology and Obstetrics Adjuvant therapy includes chemotherapy, radiotherapy and concurrent chemoradiation CT chemotherapy CCRT concurrent chemoradiation NACT neoadjuvant chemotherapy

### Associations between ADC values and clinicopathological parameters

The results of the Mann–Whitney *U* test and Student’s *t*-test for the comparison between ADC values and clinicopathological features are presented in Table 2. The ADC_mean_ of the primary tumour had a high correlation with the FIGO stage (I-IIA *vs.* IIB-IVB, 0.880 ±  0.327 *vs.* 0.655 ± 0.226, *p* = 0.001), tumour size (<4 cm *vs.* >4 cm, 0.882 ± 0.305 *vs.* 0.696 ± 0.274, *p* = 0.002), pelvic lymph node metastasis (negative *vs* positive, 0.788 ±  0.332 *vs.* 0.672 ± 0.172, *p* = 0.04), and depth of stromal invasion (inner third *vs* middle to outer third, 0.975 ± 0.378 *vs.* 0.748 ± 0.264, *p* = 0.04). The ADC_max_ of the pre-treatment tumour was greatly related to tumour size (<4 cm *vs.* >4 cm, 1.069 ± 0.269 *vs.* 0.928 ±  0.282, *p* = 0.022), FIGO stage (I-IIA *vs.* IIB-IVB, 1.068 ± 0.299 *vs.* 0.896 ± 0.252, *p* = 0.01), and depth of stromal invasion (inner third *vs* middle to outer third, 1.162 ± 0.352 *vs.* 0.951 ± 0.264, *p* = 0.023). The ADC _min_ was significantly associated with tumour size (<4 cm *vs.* >4 cm, 0.751 ± 0.335 *vs.* 0.583 ± 0.262, *p* = 0.007), FIGO stage (I-IIA *vs.* IIB-IVB, 0.761 ± 0.345 *vs.* 0.538 ±  0.206, *p* = 0.001), and pelvic lymph node metastasis (negative *vs* positive, 0.678 ± 0.329 *vs.* 0.534 ± 0.165, *p* = 0.009).

**Table 2:** ADC values and clinicopathological parameters.

Variable	*N*	ADC^mean^ (10^−3^mm^2^/s)	*P*	ADC^max^ (10^−3^mm^2^/s)	*P*	ADC^min^ (10^−3^mm^2^/s)	*P*
FIGO stage			0.001^*^		0.010^*^		0.001^*^
I-IIA	35	0.880 ± 0.327		1.068 ± 0.299		0.761 ± 0.345	
IIB-IVB	48	0.655 ± 0.226		0.896 ± 0.252		0.538 ± 0.206	
Age, years			0.630		0.380		0.628
≦45	30	0.729 ± 0.308		0.932 ± 0.306		0.612 ± 0.303	
>45	53	0.762 ± 0.287		0.989 ± 0.272		0.642 ± 0.290	
Tumor size (cm)			0.002^*^		0.022^*^		0.007^*^
<4	24	0.882 ± 0.305		1.069 ± 0.269		0.751 ± 0.335	
≧4	59	0.696 ± 0.274		0.928 ± 0.282		0.583 ± 0.262	
Pelvic LN metastasis			0.040		0.130		0.009
No	56	0.788 ± 0.332		1.002 ± 0.304		0.678 ± 0.329	
Yes	27	0.672 ± 0.172		0.901 ± 0.228		0.534 ± 0.165	
Para-aortic LN metastasis			0.408		0.193		0.346
Negative	75	0.759 ± 0.304		0.982 ± 0.292		0.642 ± 0.304	
Positive	8	0.668 ± 0.148		0.844 ± 0.165		0.538 ± 0.131	
Lymphovascular invasion			0.067		0.064		0.074
Negative	25	0.914 ± 0.347		1.108 ± 0.327		0.799 ± 0.345	
Positive	20	0.735 ± 0.278		0.934 ± 0.273		0.619 ± 0.301	
Depth of stromal invasion			0.040		0.023		0.055
Inner third	17	0.975 ± 0.378		1.162 ± 0.352		0.852 ± 0.380	
Middle to outer third	28	0.748 ± 0.264		0.951 ± 0.264		0.638 ± 0.281	
Histological homology			0.773		0.719		0.407
Pure	57	0.744 ± 0.288		0.961 ± 0.296		0.614 ± 0.294	
Mixed	26	0.764 ± 0.310		0.985 ± 0.263		0.671 ± 0.292	

**Notes.**

* *P* values were dervied applying the Mann-Whitney *U* test; other *P* values were dervied applying the Student’s *t* test.

### MRI and pathological staging of NECCs

Among the 45 patients who receiving surgery, 30 patients receiving neoadjuvant therapy were excluded, and finally, 15 patients were included in the analysis to calculate the MRI accuracy. Table 3 shows the agreement between the MRI stage and the pathological stage. The overall accuracy of MRI was only 46% (7/15). Errors were seen in eight patients due to false-negative (*n* = 2) or false-positive (*n* = 3) vaginal invasion, false-negative lymph node metastasis (*n* = 1), or false-positive parametrial invasion (*n* = 2). The accuracy rates of MRI in the diagnosis of uterine corpus invasion, parametrial invasion, vaginal invasion, and lymph node metastasis were 86.7%, 80.0%, 53.3%, and 93.3%, respectively.

**Table 3:** Comparison of MRI staging and pathological staging in surgically treated patients without neoadjuvant treatment.

Parameter	MRI	Pathology		Sensitivity	Specificity	Accuracy
		Positive	Negative			
Uterine corpus invasion	Positive	0	2	–	86.7%	86.7%
	Negative	0	13			
Parametrial invasion	Positive	0	3	–	80.0%	80.0%
	Negative	0	12			
Vaginal invasion	Positive	1	7	100.0%	50.0%	53.3%
	Negative	0	7			
Lymph node metastasis	Positive	1	0	50.0%	100.0%	93.3%
	Negative	1	13			

### Survival results

**Figure 2:**
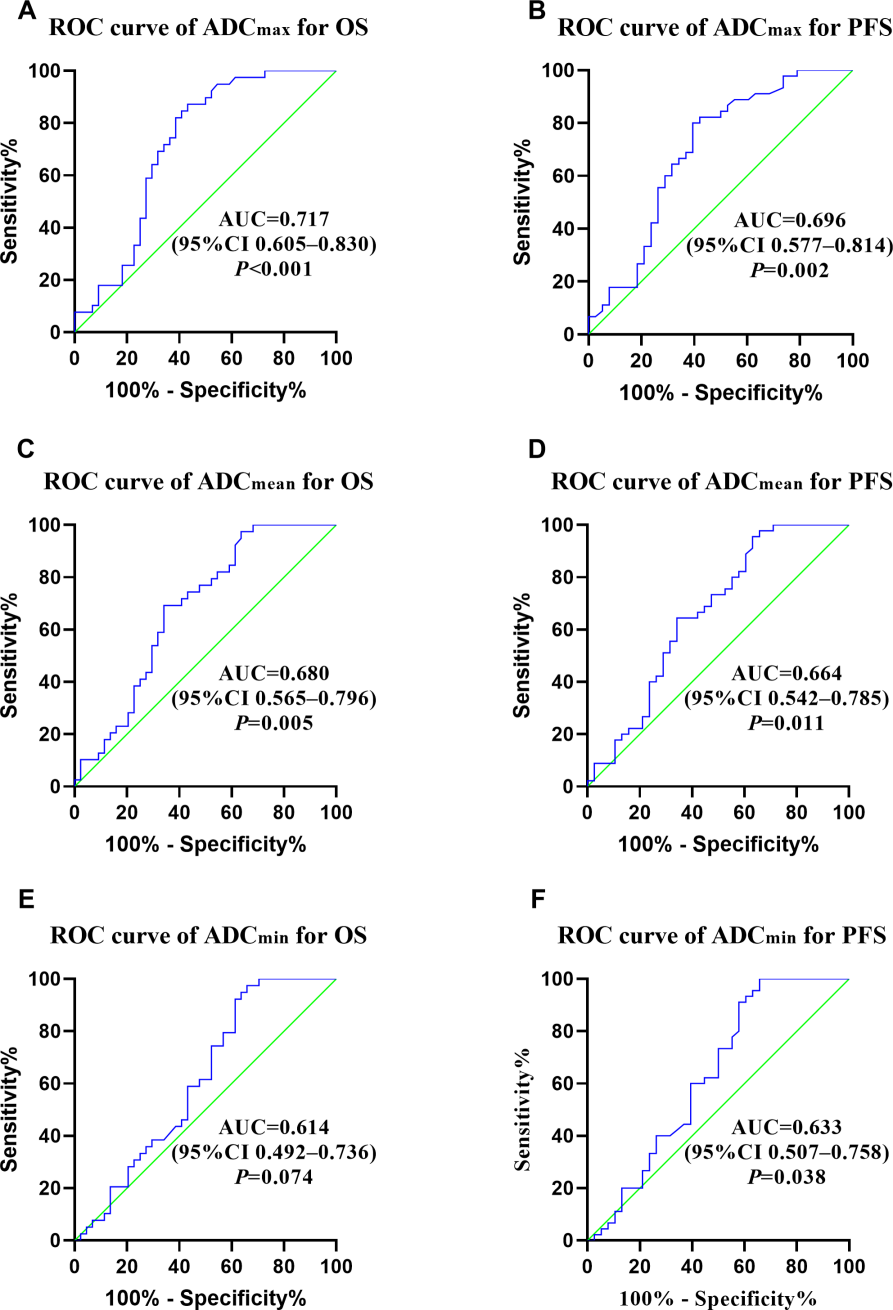
(A–F) ROC curves of ADC_max_, ADC_mean_, ADC_min_ for OS and PFS.

The median OS and PFS of the enrolled 83 patients were 42.7 and 38.1 months, and the 5-year OS and PFS rates were 46.3 and 41.4%, respectively. The median follow-up period for all patients was 50.7 months (range: 2–193 months). At the end of follow-up period, cancer recurrence was observed in 47 patients, 41 patients had died. Patients with stage I, II, III, and IV disease had 5-year OS rates of 88.9, 54.6, 35.5, and 0%, respectively. Patients with stage I, II, III, and IV disease had 5-year PFS rates of 77.8, 53.9, 21.9, and 0%, respectively. ROC curve analyses were performed to decide whether ADC values predicted the prognosis of patients diagnosed with NECCs. The optimal ADC_mean_, ADC_max_, and ADC_min_ cutoff values for OS were 0.701 × 10^−3^ mm^2^/s, 1.041 × 10^−3^ mm^2^/s, and 0.822 × 10^−3^ mm^2^/s (AUC: 0.680, 0.717 and 0.614), respectively. The optimal ADC_mean_, ADC_max_, and ADC_min_ cutoff values for PFS were 0.969 × 10^−3^ mm^2^/ss, 0.997 × 10^−3^ mm^2^/ss, and 0.922 × 10^−3^ mm^2^/s (AUC: 0.664, 0.696, and 0.633), respectively (Fig. 2).

### Prognostic factors

**Figure 3:**
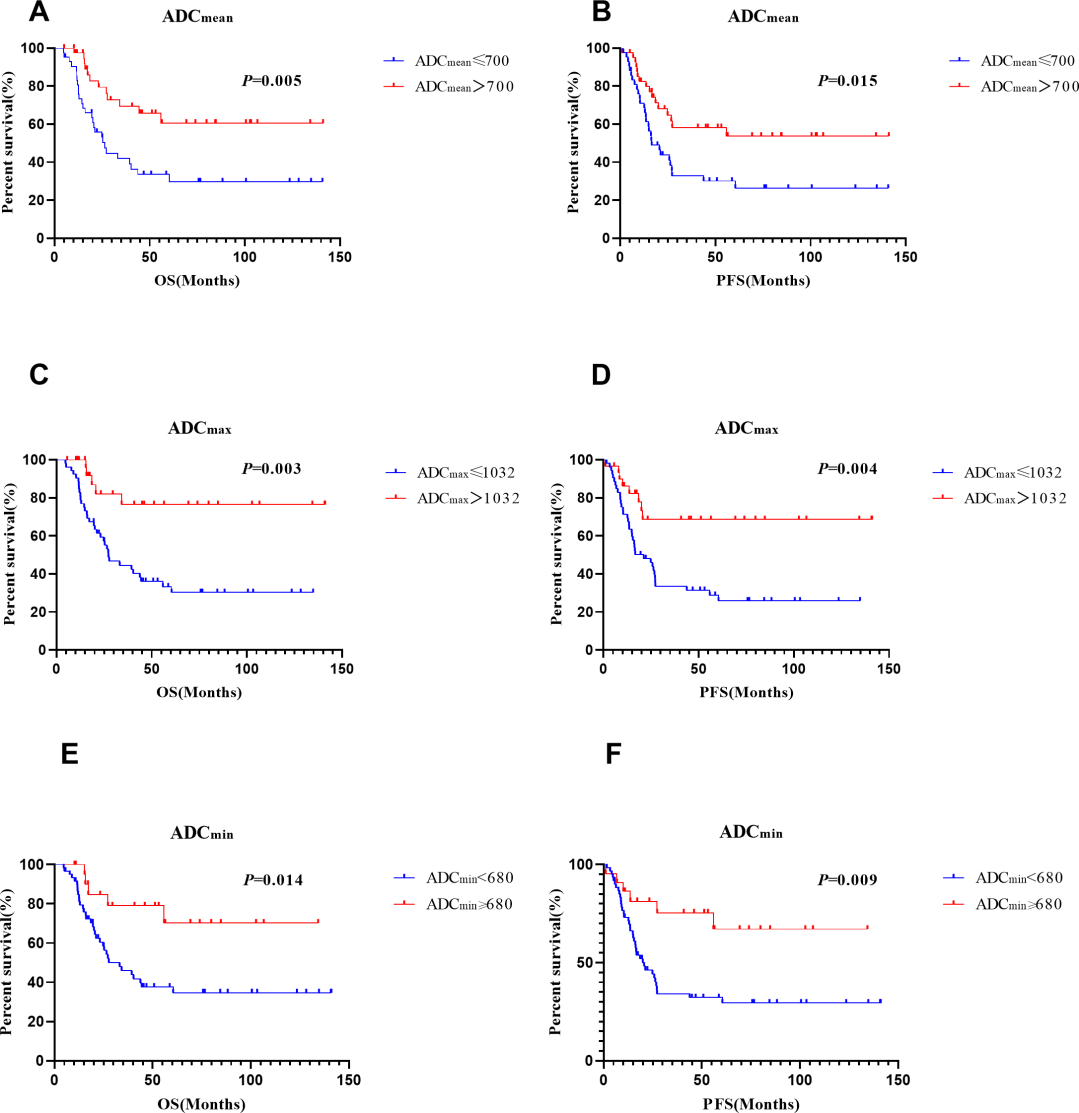
Survival curves of different ADC vaules. (A), (C) and (E) for OS, (B), (D) and (F) for PFS.

**Figure 4:**
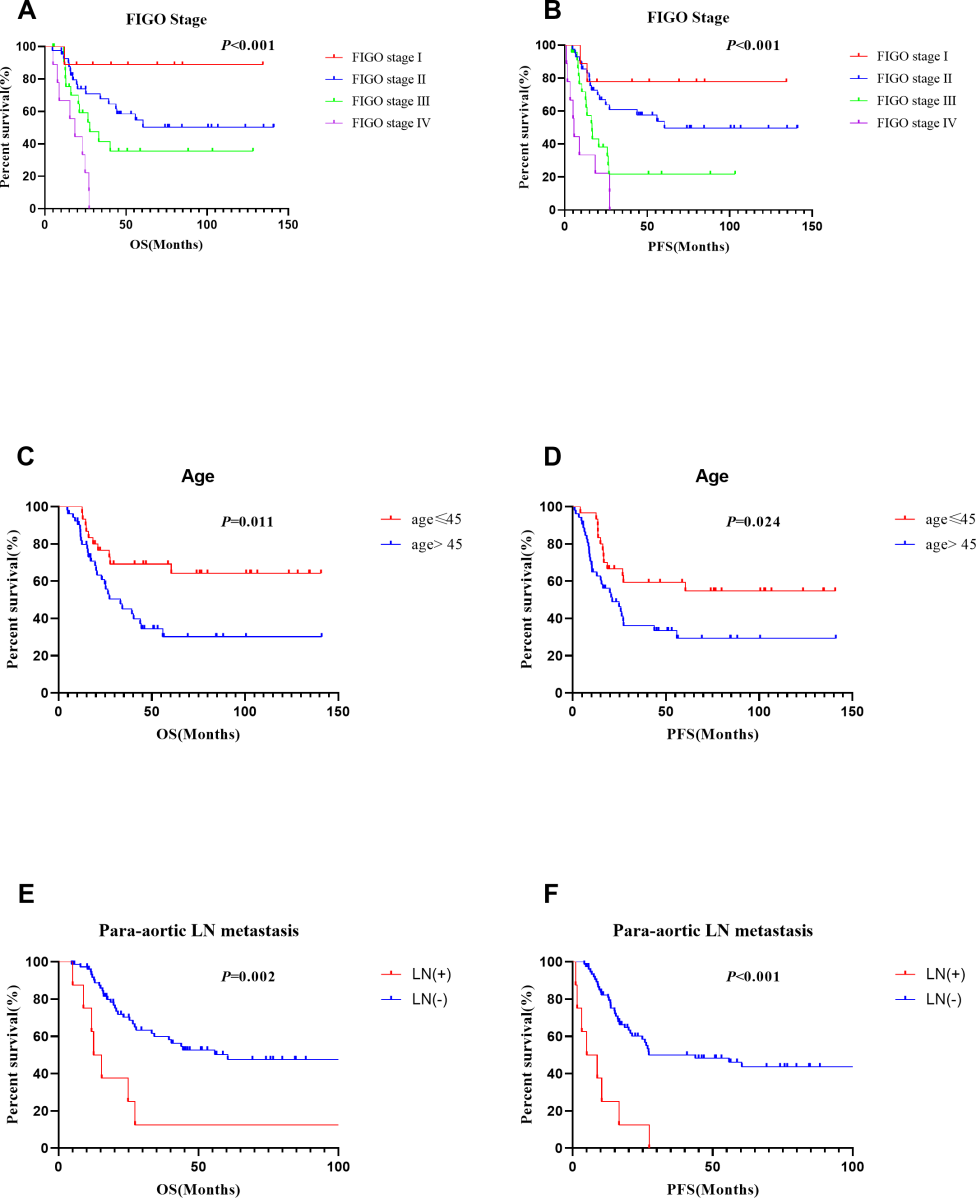
Survival curves of different FIGO stage, age and para-aortic lymph nodestatus. (A), (C) and (E) for OS, (B), (D) and (F) for PFS.

Multivariate analyses showed that ADC_mean_ (≤ 0.7 × 10^−3^ mm^2^/s *vs.* >0.7 × 10^−3^ mm^2^/s, HR =2.344, CI 95% [1.155–4.756], *p* = 0.018), advanced FIGO stage (HR =2.085, CI 95%[1.351–3.217], *p* = 0.001), and age (>45 *vs.* ≤ 45 years, HR =2.651, CI 95% [1.257–5.590], *p* = 0.01) were independent prognostic factors for OS. Besides, ADC _min_ (≤ 0.68 × 10^−3^ mm^2^/s *vs.* >0.68 × 10^−3^ mm^2^/s, HR =3.787, CI 95% [1.469–9.765], *p* = 0.006), FIGO stage (HR =1.919, CI 95% [1.221–3.014], *p* = 0.005), age (>45 *vs.* ≤ 45 years, HR =2.380, CI 95% [1.222–4.635], *p* = 0.011) , and para-aortic lymph node metastasis (positive *vs* negative, HR =3.151, CI 95% [1.204–8.248], *p* = 0.019) were significant prognostic parameters for PFS (Table 4). Survival curves for patients with various ADC values, FIGO stage, ages, and para-aortic lymph node statuses were shown in the Figs. 3 and 4. In patients who received surgery, patients with ADC_max_ >1.032 × 10^−3^ mm^2^/s had significantly better 5-year OS (92.9% *vs.* 36.9%, *p* = 0.006) and 5-year PFS (83.7% *vs.* 30.0%, *p* = 0.006) rates than those with ADC_max_ ≤ 1.032 × 10^−3^ mm^2^/s. Besides, lymphovascular invasion was another prognostic factor that affected OS (negative *vs.* positive: 76.1% *vs.* 38.3%; *p* = 0.009) (Table 5).

**Table 4:** Univariate and multivariate analysis of clinicopathological and treatment parameters for the all series (*n* = 83).

Variable	*n*	Overall survival						Progression free survival					
		Univariate			Multivariate			Univariate			Multivariate		
		HR	95%CI	*P*	HR	95%CI	*P*	HR	95%CI	*P*	HR	95%CI	*P*
Hystological type		1.537	0.369-6.406	0.555				1.424	0.344-5.893	0.626			
SCNEC	80												
LCNEC	3												
Age, years		2.552	1.236-5.270	0.011	2.651	1.257-5.590	0.01	2.117	1.106-4.054	0.024	2.380	1.222-4.635	0.011
≤45	30												
>45	53												
Tumor size(cm)		2.225	1.061-6.047	0.036	1.354	0.539-3.403	0.519	1.639	0.811-3.311	0.169			
<4	24												
≥4	59												
FIGO stage(2018)		2.339	1.578-3.469	0.001	2.085	1.351-3.217	0.001	2.423	1.634-3.342	¡0.001	1.919	1.221-3.014	0.005
I	9												
II	42												
III	23												
IV	9												
Histological homology		1.198	0.666-2.534	0.443				1.425	0.772-2.629	0.357			
Pure	57												
Mixed	26												
Pelvic LN metastasis		2.557	1.477-5.316	0.002	1.138	0.440-2.946	0.789	3.405	1.872-6.191	¡0.001	1.576	0.615-4.036	0.343
No	56												
Yes	27												
Para-aortic LN metastasis		3.287	1.573-8.203	0.002	1.265	0.439-3.649	0.663	5.241	2.405-11.422	¡0.001	3.151	1.204-8.248	0.019
No	75												
Yes	8												
Chemotherapy regimen		1.079	0.717-1.767	0.608				1.075	0.713-1.622	0.73			
TP	34												
EP	38												
Other regimens	8												
Without chemotherapy	3												
Cycle of chemotherapy		0.556	0.286-1.084	0.085	0.546	0.278-1.071	0.078	0.771	0.424-1.401	0.394			
0-5	48												
≥6	35												
ADC_min_(10^−3^mm^2^/s)		3.250	1.269-8.323	0.014	1.286	0.368-4.498	0.694	3.147	1.328-7.458	0.009	3.787	1.469-9.765	0.006
<0.680	61												
≥0.680	22												
ADC_max_(10^−3^mm^2^/s)		4.174	1.632-10.68	0.003	1.853	0.622-5.526	0.268	3.047	1.418-6.547	0.004	1.464	0.627-3.423	0.379
≤1.032	53												
>1.032	30												
ADCmean(10^−3^mm^2^/s)		2.646	1.339-5.230	0.005	2.344	1.155-4.756	0.018	2.140	1.161-3.944	0.015	1.243	0.572-2.699	0.583
≤0.700	42												
>0.700	41												

**Notes.**

Abbreviations SCNEC Small cell neuroendocrine carcinoma LCNEC large cell neuroendocrine carcinoma; Adjuvant therapy includes chemotherapy, radiotherapy and concurrent chemoradiation CT chemotherapy CCRT concurrent chemoradiation NACT neoadjuvant chemotherapy EP etoposide and cisplatin/carboplatin TP paclitaxel and cisplatin/carboplatin

**Table 5:** Univariate and multivariate analysis of clinicopathological and treatment parameters for surgically treated patients (*n* = 45).

Variable	*n*	Overall survival						Progression free survival					
		Univariate			Multivariate			Univariate			Multivariate		
		HR	95%CI	*P*	HR	95%CI	*P*	HR	95%CI	*P*	HR	95%CI	*P*
Hystological type		1.194	0.157-9.104	0.864				0.960	0.128-7.202	0.968			
SCNEC	43												
LCNEC	2												
Age, years		1.458	0.538-3.944	0.459				1.358	0.559-3.301	0.499			
≦45	21												
>45	24												
Tumor size(cm)		2.193	0.707-6.807	0.174				1.339	0.534-3.361	0.534			
<4	19												
≧4	26												
FIGO stage(2018)		2.623	1.094-6.288	0.031	0.882	0.309-2.516	0.815	2.673	1.221-5.852	0.014	0.716	0.129-3.964	0.702
I	8												
II	28												
III	9												
Histological homology		1.435	0.534-3.851	0.474				1.821	0.750-4.420	0.185			
Pure	24												
Mixed	21												
Pelvic LN metastasis		2.165	0.744-6.299	0.156				2.653	1.049-6.713	0.039	1.706	0.622-4.683	0.300
No	35												
Yes	10												
Lymphovascular invasion		4.207	1.441-12.285	0.009	3.241	1.105-9.505	0.032	3.055	1.211-7-708	0.018	1.562	0.556-4.392	0.398
Negative	25												
Positive	20												
Depth of stromal invasion		4.203	1.173-15.056	0.027	2.36	0.598-9.320	0.220	3.520	1.152-10.754	0.027	2.639	0.846-8.230	0.095
Inner third	17												
Middle to outer third	28												
Neoadjuvant therapy		1.171	0.403-3.399	0.771				1.032	0.394-2.700	0.949			
No	15												
Yes	30												
Chemotherapy regimen		0.782	0.352-1.740	0.547				0.929	0.473-1.825	0.831			
TP	17												
EP	20												
Other regimens	6												
Cycle of chemotherapy		0.48	0.173-1.334	0.159				0.747	0.293-1.903	0.54			
0-5	21												
≧6	24												
ADC_min_(10^−3^mm^2^/s)		11.49	1.515-87.163	0.018	1.440	0.096-21.552	0.792	6.983	1.616-30.173	0.009	2.084	0.287-15.12	0.468
<0.680	28												
≧0.680	17												
ADC_max_(10^−3^mm^2^/s)		16.69	2.230-128.977	0.006	14.413	1.883-110.342	0.01	6.667	1.945-22.850	0.003	5.668	1.634-19.66	0.006
≦1.032	24												
>1.032	21												
ADC_mean_(10 −3mm^2^/s)		4.194	1.447-12.160	0.008	1.257	0.345-4.578	0.729	2.740	1.118-6.717	0.028	3.655	0.639-20.91	0.145
≦0.700	18												
>0.700	27												

**Notes.**

Abbreviations SCNEC Small cell neuroendocrine carcinoma LCNEC large cell neuroendocrine carcinoma Adjuvant therapy includes chemotherapy, radiotherapy and concurrent chemoradiation CT chemotherapy CCRT concurrent chemoradiation NACT neoadjuvant chemotherapy EP etoposide and cisplatin/carboplatin TP paclitaxel and cisplatin/carboplatin.

## Discussion

To the best of our knowledge, this study was the first to investigate the prognostic utility of ADC values in predicting the outcomes of patients with NECCs. Moreover, the correlation between the ADC values and clinicopathological parameters in neuroendocrine carcinomas has been reported for the first time. We also assessed the accuracy of MRI in the diagnosis of NECCs. It was previously suggested that the decreased ADC values in malignant tumours indicated proliferative activity and increased tissue cellularity, which led to the disordered arrangement of the intracellular structure and decreased extracellular spaces (Nakamura et al., 2012; Schob et al., 2017b). Additionally, some studies have reported that ADC values reflect the tumour aggressiveness and predicted prognosis and treatment response to chemoradiation therapy. De Robertis et al. (2018) found that ADC maps may help predict the tumour grade, vascular involvement, and nodal and liver metastases in pancreatic neuroendocrine tumours. Heo et al. (2013) showed that pre-treatment ADC values could predict the tumour recurrence in patients who were diagnosed with cervical cancer and treated with chemoradiation. They found that patients with the 75th percentile ADC >0.936 × 10^−3^ mm^2^/s had significantly better overall recurrence free survival raterates than those with the 75th percentile ADC ≤0.936 × 10^−3^ mm^2^/s (91.7% *vs.* 51.9%, *p* = 0.003) . Other researches have also shown that ADC analysis may be an effective clinical biomarker to forecast treatment response and survival rate among patients with hepatocellular carcinoma and rectal cancer (Choi et al., 2016; Shaghaghi et al., 2020). However, the efficacy of ADC values in predicting FIGO staging and prognosis in patients with NECCs is unclear.

Therefore, we explored whether pre-treatment ADC values were associated with clinicopathological characteristics in patients with NECC in this study. It was observed that lower ADC values were greatly associated with advanced FIGO stage, large tumour size, deep stromal invasion, and pelvic lymph node metastasis. We used the pre-treatment MRI and demonstrated that ADC values were greatly related to the prognosis in patients with NECCs. In multivariate analysis, ADC_mean_ ≤ 0.7 × 10^−3^ mm^2^/s were associated with wores overall survival rates (HR =2.344, *p* = 0.018) and ADC _min_ ≤ 0.68 × 10^−3^ mm^2^/s were associated with worse progression-free survival rates (HR =3.787, *p* = 0.006). These findings are similar to those of a prior study. Zhao et al. reported that pre-treatment ADC_min_ was significantly correlated with the disease-free survival in patients with cervical cancer (HR = 0.110, *p* = 0.006) (Zhao et al., 2019). In surgically treated patients, we found that ADC _max_ ≤ 1.032 × 10^−3^ mm^2^/s was greatly associated with worse OS and PFS. The risk of recurrence and disease progression increased by 14.4 and 5.7 times, respectively, compared with those with ADC _max_>1.032 × 10^−3^ mm^2^/s. ROC curve analyses were performed to decide which ADC value among these three values performing the best ability. ADC _max_ seems to have better diagnostic effectiveness than ADC_mean_ and ADC_min_, and the AUC of ADC_max_ for OS and PFS was 0.717 and 0.696, respectively.

This research showed that FIGO stage and age at diagnosis were prognostic factors for OS, and FIGO stage, age at diagnosis, and para-aortic lymph node metastasis were prognostic parameters for PFS; these results are similar to those of our previous study (Chen et al., 2021). Additionally, the lymphovascular invasion was founded to be a prognostic parameter for 5-year OS in the patients who underwent surgery; however, FIGO stage was not. This may be due to the fact that the proportion of lymphovascular invasion in patients with stage I disease (50.0%) was higher than that in patients with stage II disease (32.1%).

MRI is regarded as a useful and accurate method for the diagnosis of cervical tumours. Sala et al. (2007) found that MRI was 83% (8/41) accurate in diagnosing cervical carcinomas with vaginal invasion. They assumed that this inaccuracy was caused by a large exophytic cervical tumour stretching the vaginal fornix. A meta-analysis of 57 studies showed that the sensitivity for parametrial invasion in MRI was 74% (Bipat et al., 2003). In our study, the accuracy rates of MRI in the diagnosis of uterine corpus invasion, parametrial invasion, vaginal invasion, and lymph node metastasis were 86.7%, 80.0%, 53.3%, and 93.3%, respectively. There were two false-positive parametrial invasions. Cervical biopsy or cervical conisation was performed before MRI examination, resulting in inflammation and stromal oedema; this resulted in an inadequate estimation of parametrial invasion. Stromal oedema caused by tumour compression is also a possible cause (Nakamura et al., 2012; Park et al., 2014). Additionally, Woo et al. (2019) found that for determining parametrial invasion, oblique axial T2WI may be more accurate than true axial T2WI, especially for tumours larger than 2.5 cm.

According to a meta-analysis of 72 studies comprising 5,042 patients, MRI exhibited a sensitivity of 56% and a specificity of 93% for detecting lymphadenopathy (Choi et al., 2010). For detecting lymph node metastasis, the size criterion used in MRI was a short axis diameter ≥ one cm (Balleyguier et al., 2011; Dappa et al., 2017). This criterion, however, is flawed because it overlaps with normal, hyperplastic, and metastatic lymph nodes. Furthermore, micrometastases in negative lymph nodes are not rare (Lee, Kim & Park, 2020). Therefore, we considered round shape, irregular border, and necrosis as other signs of malignancy (Balleyguier et al., 2011). In our study, the sensitivity, specificity and accuracy of MRI in detecting lymph node metastasis were 50.0%, 100%, and 93.3%, respectively. Lin et al. (2008) observed that the method combining tumour size and ADC values had better sensitivity (25% *vs.* 83%) and similar specificity (98% *vs.* 99%) compared with those of the traditional MRI approach. MRI was useful in detecting the parametrial invasion, uterine corpus invasion, and lymph node metastasis; however, the diagnostic efficacy of vaginal invasion needs to be improved.

There are several limitations in this research. First, this was a retrospective study; therefore, selection bias was unavoidable. Second, we did not measure ADC values for the entire tumour in this study. Further studies using histogram analyses will be needed. Moreover, ROIs were drawn manually by two radiologists, and measurement errors were inevitable. Third, the study did not include dynamic contrast-enhanced MRI, which is a useful diagnostic tool. Fourth, para-aortic lymph node metastasis affected OS in our research as an independent prognostic parameter; however; only 22% (10/45) of the patients had para-aortic lymph node dissection. Finally, this was a single-centre study with a small sample size, especially for those with LCNECs. More studies involving larger cohorts are needed.

## Conclusion

It was observed that lower ADC values were greatly associated with advanced FIGO stage, large tumour size, deep stromal invasion, and lymph node metastasis. ADC_mean_ and ADC _min_ were independent prognostic parameters for NECCs. For surgically treated patients (*n* = 45), ADC _max_ was an independent prognostic parameter for both 5-year OS and PFS. Additionally, we found that MRI is reliable for the prediction of uterine corpus invasion, parametrial invasion, and lymph node metastasis, but not vaginal invasion. ADC analysis may be a useful tool for predicting the FIGO stage and outcome of patients with NECCs.

##  Supplemental Information

10.7717/peerj.15084/supp-1 Supplemental Information 1 Raw data

## Additional Information and Declarations

### Data Availability

The following information was supplied regarding data availability: The raw measurements are available in the Supplementary File.

## References

[ref-1] Balleyguier C, Sala E, Da Cunha T, Bergman A, Brkljacic B, Danza F, Forstner R, Hamm B, Kubik-Huch R, Lopez C, Manfredi R, McHugo J, Oleaga L, Togashi K, Kinkel K (2011). Staging of uterine cervical cancer with MRI: guidelines of the European Society of Urogenital Radiology. European Radiology.

[ref-2] Bhatla N, Aoki D, Sharma DN, Sankaranarayanan R (2018). Cancer of the cervix uteri. International Journal of Gynecology & Obstetrics.

[ref-3] Bhatla N, Berek JS, Cuello Fredes M, Denny LA, Grenman S, Karunaratne K, Kehoe ST, Konishi I, Olawaiye AB, Prat J, Sankaranarayanan R, Brierley J, Mutch D, Querleu D, Cibula D, Quinn M, Botha H, Sigurd L, Rice L, Ryu HS, Ngan H, Maenpaa J, Andrijono A, Purwoto G, Maheshwari A, Bafna UD, Plante M, Natarajan J (2019). Revised FIGO staging for carcinoma of the cervix uteri. International Journal of Gynecology & Obstetrics.

[ref-4] Bipat S, Glas AS, van der Velden J, Zwinderman AH, Bossuyt PM, Stoker J (2003). Computed tomography and magnetic resonance imaging in staging of uterine cervical carcinoma: a systematic review. Gynecologic Oncology.

[ref-5] Bruix J, Llovet JM (2002). Prognostic prediction and treatment strategy in hepatocellular carcinoma. Hepatology.

[ref-6] Chen J, Sun Y, Chen L, Zang L, Lin C, Lu Y, Lin L, Lin A, Dan H, Chen Y, He H (2021). Prognostic factors and treatment of neuroendocrine tumors of the uterine cervix based on the FIGO 2018 staging system: a single-institution study of 172 patients. PeerJ.

[ref-7] Choi HJ, Ju W, Myung SK, Kim Y (2010). Diagnostic performance of computer tomography, magnetic resonance imaging, and positron emission tomography or positron emission tomography/computer tomography for detection of metastatic lymph nodes in patients with cervical cancer: meta-analysis. Cancer Science.

[ref-8] Choi MH, Oh SN, Rha SE, Choi JI, Lee SH, Jang HS, Kim JG, Grimm R, Son Y (2016). Diffusion-weighted imaging: apparent diffusion coefficient histogram analysis for detecting pathologic complete response to chemoradiotherapy in locally advanced rectal cancer. Journal of Magnetic Resonance Imaging.

[ref-9] Dappa E, Elger T, Hasenburg A, Duber C, Battista MJ, Hotker AM (2017). The value of advanced MRI techniques in the assessment of cervical cancer: a review. Insights Imaging.

[ref-10] De Robertis R, Maris B, Cardobi N, Tinazzi Martini P, Gobbo S, Capelli P, Ortolani S, Cingarlini S, Paiella S, Landoni L, Butturini G, Regi P, Scarpa A, Tortora G, D’Onofrio M (2018). Can histogram analysis of MR images predict aggressiveness in pancreatic neuroendocrine tumors?. European Radiology.

[ref-11] Duan X, Ban X, Zhang X, Hu H, Li G, Wang D, Wang CQ, Zhang F, Shen J (2016). MR imaging features and staging of neuroendocrine carcinomas of the uterine cervix with pathological correlations. European Radiology.

[ref-12] Fan C, Min X, Feng Z, Cai W, Li B, Zhang P, You H, Xie J, Wang L (2020). Discrimination between benign and malignant testicular lesions using volumetric apparent diffusion coefficient histogram analysis. European Journal of Radiology.

[ref-13] Gadducci A, Carinelli S, Aletti G (2017). Neuroendrocrine tumors of the uterine cervix: a therapeutic challenge for gynecologic oncologists. Gynecologic Oncology.

[ref-14] Gardner GJ, Reidy-Lagunes D, Gehrig PA (2011). Neuroendocrine tumors of the gynecologic tract: a Society of Gynecologic Oncology (SGO) clinical document. Gynecologic Oncology.

[ref-15] Gu KW, Kim CK, Choi CH, Yoon YC, Park W (2019). Prognostic value of ADC quantification for clinical outcome in uterine cervical cancer treated with concurrent chemoradiotherapy. European Radiology.

[ref-16] Heo SH, Shin SS, Kim JW, Lim HS, Jeong YY, Kang WD, Kim SM, Kang HK (2013). Pre-treatment diffusion-weighted MR imaging for predicting tumor recurrence in uterine cervical cancer treated with concurrent chemoradiation: value of histogram analysis of apparent diffusion coefficients. Korean Journal of Radiology.

[ref-17] Lee J, Kim CK, Park SY (2020). Histogram analysis of apparent diffusion coefficients for predicting pelvic lymph node metastasis in patients with uterine cervical cancer. MAGMA.

[ref-18] Li J, Ouyang Y, Tao Y, Wang L, Li M, Gao L, Cao X (2020). Small cell carcinoma of the uterine cervix: a multi-institutional experience. International Journal of Gynecologic Cancer.

[ref-19] Liang HY, Huang YQ, Yang ZX, Ying D, Zeng MS, Rao SX (2016). Potential of MR histogram analyses for prediction of response to chemotherapy in patients with colorectal hepatic metastases. European Radiology.

[ref-20] Lin G, Ho KC, Wang JJ, Ng KK, Wai YY, Chen YT, Chang CJ, Ng SH, Lai CH, Yen TC (2008). Detection of lymph node metastasis in cervical and uterine cancers by diffusion-weighted magnetic resonance imaging at 3T. Journal of Magnetic Resonance Imaging.

[ref-21] Lin LM, Lin Q, Liu J, Chu KX, Huang YX, Zhang ZK, Li T, Dai YQ, Li JL (2020). Prognostic factors and treatment comparison in small cell neuroendocrine carcinoma of the uterine cervix based on population analyses. Cancer Medicine.

[ref-22] Liu S, Guan W, Wang H, Pan L, Zhou Z, Yu H, Liu T, Yang X, He J, Zhou Z (2014). Apparent diffusion coefficient value of gastric cancer by diffusion-weighted imaging: correlations with the histological differentiation and Lauren classification. European Journal of Radiology.

[ref-23] McCann GA, Boutsicaris CE, Preston MM, Backes FJ, Eisenhauer EL, Fowler JM, Cohn DE, Copeland LJ, Salani R, O’Malley DM (2013). Neuroendocrine carcinoma of the uterine cervix: the role of multimodality therapy in early-stage disease. Gynecologic Oncology.

[ref-24] Meyer HJ, Gundermann P, Hohn AK, Hamerla G, Surov A (2019). Associations between whole tumor histogram analysis parameters derived from ADC maps and expression of EGFR, VEGF, Hif 1-alpha, Her-2 and Histone 3 in uterine cervical cancer. Magnetic Resonance Imaging.

[ref-25] Nakamura K, Joja I, Nagasaka T, Fukushima C, Kusumoto T, Seki N, Hongo A, Kodama J, Hiramatsu Y (2012). The mean apparent diffusion coefficient value (ADCmean) on primary cervical cancer is a predictive marker for disease recurrence. Gynecologic Oncology.

[ref-26] Park JJ, Kim CK, Park SY, Park BK, Kim B (2014). Value of diffusion-weighted imaging in predicting parametrial invasion in stage IA2-IIA cervical cancer. European Radiology.

[ref-27] Perucho JAU, Wang M, Tse KY, Ip PPC, Siu SWK, Ngan HYS, Khong PL, Lee EYP (2020). Association between MRI histogram features and treatment response in locally advanced cervical cancer treated by chemoradiotherapy. European Radiology.

[ref-28] Sala E, Wakely S, Senior E, Lomas D (2007). MRI of malignant neoplasms of the uterine corpus and cervix. AJR American Journal of Roentgenology.

[ref-29] Satoh T, Takei Y, Treilleux I, Devouassoux-Shisheboran M, Ledermann J, Viswanathan AN, Mahner S, Provencher DM, Mileshkin L, Avall-Lundqvist E, Pautier P, Reed NS, Fujiwara K (2014). Gynecologic Cancer InterGroup (GCIG) consensus review for small cell carcinoma of the cervix. International Journal of Gynecologic Cancer.

[ref-30] Schob S, Meyer HJ, Dieckow J, Pervinder B, Pazaitis N, Hohn AK, Garnov N, Horvath-Rizea D, Hoffmann KT, Surov A (2017a). Histogram analysis of diffusion weighted imaging at 3T is useful for prediction of lymphatic metastatic spread, proliferative activity, and cellularity in thyroid cancer. International Journal of Molecular Sciences.

[ref-31] Schob S, Meyer HJ, Pazaitis N, Schramm D, Bremicker K, Exner M, Hohn AK, Garnov N, Surov A (2017b). ADC histogram analysis of cervical cancer aids detecting lymphatic metastases-a preliminary study. Molecular Imaging and Biology.

[ref-32] Shaghaghi M, Aliyari Ghasabeh M, Ameli S, Ghadimi M, Hazhirkarzar B, Rezvani Habibabadi R, Khoshpouri P, Pandey A, Pandey P, Kamel IR (2020). Post-TACE changes in ADC histogram predict overall and transplant-free survival in patients with well-defined HCC: a retrospective cohort with up to 10 years follow-up. European Radiology.

[ref-33] Woo S, Moon MH, Cho JY, Kim SH, Kim SY (2019). Diagnostic performance of MRI for assessing parametrial invasion in cervical cancer: a head-to-head comparison between oblique and true axial T2-weighted images. Korean Journal of Radiology.

[ref-34] Yuan L, Jiang H, Lu Y, Guo SW, Liu X (2015). Prognostic factors of surgically treated early-stage small cell neuroendocrine carcinoma of the cervix. International Journal of Gynecologic Cancer.

[ref-35] Zhao B, Cao K, Li XT, Zhu HT, Sun YS (2019). Whole lesion histogram analysis of apparent diffusion coefficients on MRI predicts disease-free survival in locally advanced squamous cell cervical cancer after radical chemo-radiotherapy. BMC Cancer.

[ref-36] Zou X, Luo Y, Li Z, Hu Y, Li H, Tang H, Shen Y, Hu D, Kamel IR (2019). Volumetric apparent diffusion coefficient histogram analysis in differentiating intrahepatic mass-forming cholangiocarcinoma from hepatocellular carcinoma. Journal of Magnetic Resonance Imaging.

